# An Anti-Freezing Ionic Conductive Hydrogel for Strain Sensing and Energy Harvesting Devices

**DOI:** 10.3390/polym17233102

**Published:** 2025-11-22

**Authors:** Yanjie Wang, Wei Yu, Sijun Liu

**Affiliations:** Advanced Rheology Institute, Department of Polymer Science and Engineering, Shanghai Jiao Tong University, Shanghai 200240, China

**Keywords:** anti-freezing ionic conductive hydrogels, physical double network, strain sensor, triboelectric nanogenerator

## Abstract

Hydrogels with excellent flexibility and conductivity have attracted intensive attention in wearable human monitoring and energy harvesting devices. However, hydrogels containing plenty of water inevitably freeze at subzero temperatures, which deteriorates flexibility and conductivity and limits their practical applications. Herein, an anti-freezing ionic conductive hydrogel is developed by introducing Na^+^ into the gellan gum/hydrophobically associated polyacrylamide double network. The optimized anti-freezing hydrogel AICH_3_ achieves outstanding mechanical properties (fracture stress 1.1 MPa and fracture strain 1700%), remarkable conductivity (2.2 S/m), and impressive strain sensitivity (GF = 7.4) at −20 °C. Benefiting from excellent flexibility, conductivity and strain sensitivity, the assembled AICH_3_-based strain sensor can accurately sense the bending movement of the bionic finger at −20 °C. In addition, the AICH_3_ can also be used as a stretchable electrode of a triboelectric nanogenerator (TENG), and the assembled AICH_3_-based TENG can effectively harvest energy and power electronic devices at −20 °C. The comprehensive mechanical and conductive properties of AICH_3_ at subzero temperatures might be attributed to the multifunctionality of Na^+^, which not only promotes the fabrication of physically crosslinked gellan gum/hydrophobically associated polyacrylamide double network but also suppresses the formation of ice crystals.

## 1. Introduction

In recent years, flexible electronics have received increasing interest due to their extensive applications in the fields of human-machine interaction [1,2,3], smart electronic skin [4,5,6], implantable medical devices [7,8,9], and energy harvesting devices [10,11,12]. As a key component of flexible electronic devices, a stretchable conductor with remarkable strain sensitivity and stability plays a major role [13,14]. At present, most conductors for flexible devices are made by adding intrinsically conductive materials (e.g., carbon nanotubes (CNTs) [15,16], graphene [17,18], metallic nanoparticles (NPs) [19,20], etc.) or introducing conductive polymers (e.g., polyaniline (PANI) [21,22,23], polypyrrole (PPy) [24,25,26], poly(3,4-ethylenedioxythiophene) (PEDOT) [27,28,29], etc.) into a stretchable matrix, such as elastomer and rubber. However, these flexible devices usually show limited flexibility, a narrow sensing range, and a lack of long-term stability, thus limiting their widespread applications.

Conductive hydrogels are able to detect and respond to external stimuli and transform mechanical deformation into electrical signals, drawing intensive attention to flexible devices. Recently, the progress of stretchable and tough hydrogels has greatly promoted the development of conductive hydrogels in wearable electronic devices [14,30,31,32,33]. However, hydrogels containing plenty of water are inevitably frozen, which makes polymer chains difficult to move, thus resulting in invalid hydrogel devices with failed mechanical flexibility and negligible conductivity. Currently, various strategies have been developed to inhibit the formation of ice crystalline and obtain anti−freezing hydrogels. One is adding anti−freezing solvents, such as glycerol, ethylene glycol, sorbitol, or a mixture of them, into hydrogel [34,35]. Although anti-freezing solvents can effectively weaken the hydrogen bonding interaction of water molecules and prevent the formation of ice crystals, these organic solvents usually cause adverse effects on the conductivity of hydrogels and have potential harm to human health and the environment. The second strategy is introducing salts into hydrogels to inhibit the formation of ice crystals, and the presence of salts could enhance the conductivity of hydrogels [36,37,38]. However, high concentrations of ions might weaken the mechanical performance of hydrogels, owing to the oversaturated interaction (hydrogen bonding, ionic bonding, etc.) inside the network. Therefore, it is still challenging to fabricate conductive hydrogels with comprehensive mechanical properties, freezing tolerance and conductivity at subzero temperatures.

Herein, we prepared an anti-freezing ionic conductive hydrogel (AICH_3_) by introducing Na^+^ into the gellan gum/acrylamide aqueous solution. The presence of Na^+^ has three purposes: the first is to construct the physical double network, including the hydrophobically associated polyacrylamide (HAP) network and the ionic crosslinked gellan gum (GG) network to improve the mechanical performance of the hydrogel; the second is to provide the hydrogel with excellent ionic conductivity; and the third is to endow the hydrogel with outstanding anti-freezing capability. During the deformation process, both hydrophobic micelles and GG aggregates serve as reversible “sacrifice bonds” to consume energy, which leads to the resultant hydrogels achieving fracture stress 0.7 MPa, fracture strain 4500%, ionic conductivity 9.1 S/m, and freezing tolerance as low as −53 °C. More importantly, the AICH_3_ also demonstrates fracture stress 1.1 MPa, fracture strain 1700%, fatigue resistance 1000 cycles, conductivity 2.2 S/m, and strain sensitivity GF = 7.4 at −20 °C. The comprehensive properties of AICH_3_ ensure that the assembled AICH_3_-based strain sensor can accurately sense the movement of the bionic finger at −20 °C. In addition, the AICH_3_ can also be used as a stretchable electrode material of TENG, and the assembled AICH_3_-based TENG can effectively harvest energy and power electronic devices at −20 °C. Overall, the AICH_3_ shows an application potential in flexible devices at subzero temperatures.

## 2. Experimental Section

### 2.1. Materials

Low acyl gellan gum (GG) with the weight-average molecular weight (M_w_) of 6.0 × 10^5^ was provided by DSM Zhongken Biotechnology Co., Ltd., Tongxiang, China. The contents of metallic ions of Na^+^, K^+^, Ca^2+^, and Mg^2+^ are 0.429%, 3.917%, 0.241%, and 0.097%, respectively, measured by the inductively coupled plasma (ICP) (iCAP 6000 ICP, Thermo, Inc., Cambridge, UK). Acrylamide (AAm, 99%) was provided by Macklin Biotechnology Co., Ltd., Shanghai, China. Stearyl methacrylate (SMA, 95%) and 2-hydroxy-4’-(2-hydroxyethoxy)-2-methylpropiophenone (I2959, 98%) were purchased from Sigma Aldrich, Shanghai, China. Sodium chloride (NaCl, ≥99.5%) and sodium dodecyl sulfate (SDS, >85%) were obtained from Aladdin Biotechnology Co., Ltd., Shanghai, China. All of the reagents were used as received.

### 2.2. Synthesis of AICH

The anti-freezing ionic conductive hydrogels (AICH_x_) were prepared through the one-pot method, where x represents the content of NaCl (mmol/g). Taking AICH_3_ as an example, GG powders (1.67 × 10^−4^ mmol), AAm (77.1 mmol) and SMA monomers (1.54 mmol) were first added into a reaction flask containing deionized water (6 g) and stirred continuously at 85 °C for 2 h. Then, photo-initiator I2959 (0.39 mmol), SDS (4.85 mmol) and NaCl solution (6 mmol/g) were slowly added into the above aqueous solution and continuously stirred at 85 °C for 5 h. Subsequently, the obtained solution was poured into the mold and UV curing (wavelength = 365 nm, density = 400 W) for 5 min to form the hydrophobically associated PAAm network. Finally, the AICH were obtained through cooling at room temperature for 2 h.

### 2.3. Microstructures

The microstructures of hydrogels were obtained by a field emission scanning electron microscope (Nova Nano FESEM 450 from FEI, Hillsboro, OR, USA). The samples for FESEM were prepared as follows: the AICH were immersed in liquid nitrogen for 5 min, and then moved quickly to the lyophilizer for freeze-drying. The freeze-dried gels were fractured in liquid nitrogen. The cross-sections were sprayed with gold in a vacuum and examined by FESEM at an accelerating voltage of 10 kV.

### 2.4. Anti-Freezing Characteristics

The freezing tolerance of hydrogels was first characterized by measuring the freezing point based on Differential Scanning Calorimetry (TA DSC Q2000, New Castle, DE, USA). The temperature range is from 40 °C to −80 °C at a cooling rate of 10 °C/min. Meanwhile, the rheological temperature sweep (TA ARES-G2, New Castle, DE, USA) was also carried out to determine the anti-freezing property, where the phase angle tanδ is represented as a function of temperature.

### 2.5. Mechanical Property

The mechanical property of AICH with a dumbbell shape (a width of 5 mm in the middle, a thickness of 2 mm and a length of 50 mm) was tested at a speed of 100 mm/min by the WDW-GD universal testing machine (Shanghai Songton Instrument Co., Ltd., Shanghai, China). The mechanical property at subzero temperatures was also carried out on the universal testing machine within a low−temperature chamber. Each sample was kept at the testing temperature for 30 min to achieve a thermal equilibrium before testing.

### 2.6. Conductivity

The conductivity of AICH was measured by a DCR meter (UC2856, Youce Electronic Technology Co., Ltd., Changzhou, China) at an alternating current with a voltage of 1 V and a frequency of 100 kHz. The relative resistance change, $∆R/R_{0}=(R-R_{0})/R_{0},$ was determined, where $R_{0}$ is the initial resistance and *R* is the real-time resistance. The gauge factor (GF) was calculated from the slope of the relative resistance change ($∆R/R_{0}$) versus strain.

### 2.7. Fabrication of AICH-Based Devices

The PDMS film was first prepared by mixing the pre-polymer with the curing agent at a ratio of 10:1 and then casting it into the mold at a thickness of 1 mm, followed by curing for 5 h at 60 °C. The AICH-based TENG was fabricated by assembling the AICH and two pieces of PDMS films into the sandwich structure, where the AICH acts as the electrode and the PDMS films act as the positive electrification layer and the support substrate. The copper wire was connected to the AICH for electrical output measurements. The output performance (Voc, Isc, Qsc) of AICH-based TENG was measured by an electrometer (Keithley 6514, Cleveland, OR, USA).

## 3. Results and Discussion

### 3.1. Preparation of AICH

**Figure 1:**
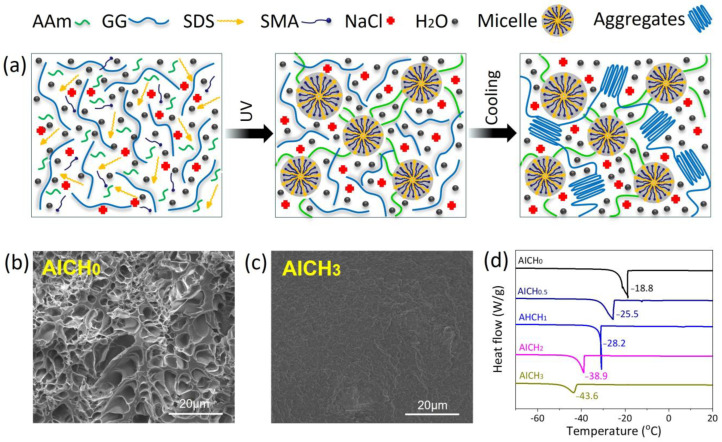
(**a**) Schematic diagram of AICH preparation. FESEM images of AICH with various NaCl concentrations, (**b**) 0 mmol/g and (**c**) 3 mmol/g. (**d**) DSC curves of AICH with various NaCl contents.

The schematic diagram for the preparation of AICH is presented in Figure 1a. The hydrophobic SMA monomers are dissolved in the self-assembled SDS micelles under the solubilization of NaCl, and then the HAP network is formed under UV light, where the SMA micelles are used as the hydrophobic association point. As the temperature is decreased, the conformation of GG changes from random coils to double helices, and then the aggregation of double helices results in the formation of the GG network. Finally, the physically crosslinked GG/HAP double network is obtained. In the GG/HAP double network hydrogel, NaCl plays multiple roles. First of all, NaCl increases the solubility of hydrophobic SMA in aqueous solution and promotes the formation of micelles and construction of HAP; NaCl also enhances the aggregation capability of gellan gum double helices, thus increasing the crosslinking densities of HAP and GG and producing a dense network, as evidenced by FESEM images of HAP/GG hydrogels (Figure 1b,c). It is apparent that the HAP/GG hydrogels with a high content of NaCl (3 mmol/g) produce a denser network structure than those without NaCl. The second is that the presence of NaCl weakens the hydrogen bonding interaction of H_2_O molecules, thus greatly decreasing the freezing point of GG/HAP hydrogel. It is found from DSC measurements that the freezing point decreases from −18.8 °C to −43.4 °C as the content of NaCl increases from 0 mmol/g to 3 mmol/g (Figure 1d). The rheological temperature sweep demonstrates a similar freezing tolerance of GG/HAP hydrogels. The third is that the presence of NaCl greatly enhances the conductivity of the resultant GG/HAP double network hydrogel at subzero temperatures, which will be discussed later.

### 3.2. Mechanical Property of AICH

**Figure 2:**
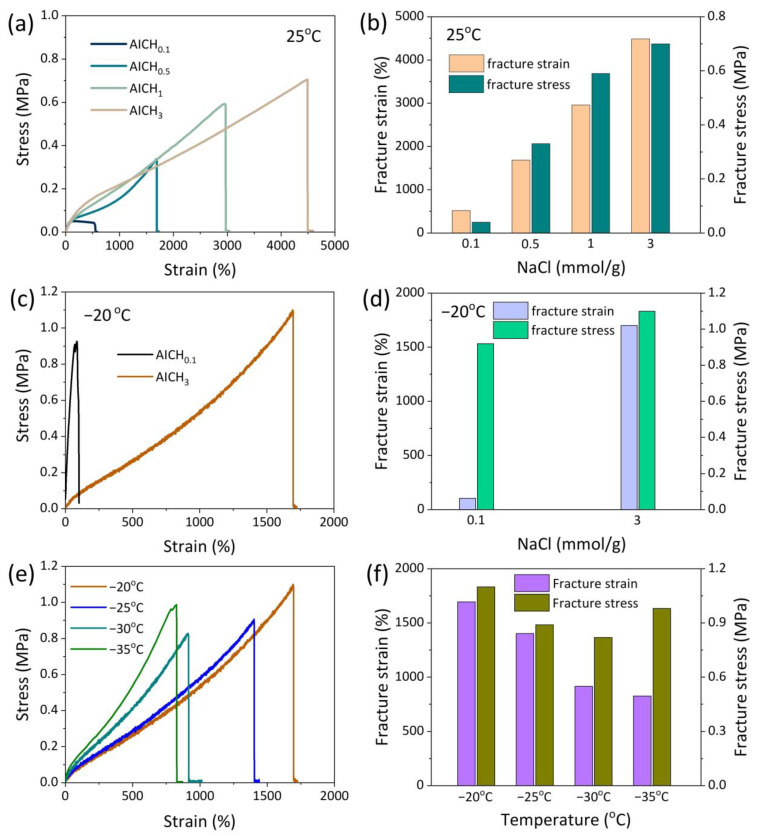
(**a**) The tensile stress-strain curves of AICH with various NaCl contents at room temperature, and (**b**) the corresponding effect of NaCl on the fracture strain and stress. (**c**) The tensile stress−strain curves of hydrogels with various NaCl contents at −20 °C, and (**d**) the corresponding effect of NaCl on the fracture strain and stress. (**e**) The tensile stress-strain curves of AICH_3_ at different subzero temperatures, and (**f**) the corresponding effect of temperature on the fracture strain and stress.

The mechanical properties of AICH at room temperature (25 °C) are first evaluated by tensile measurements. The presence of NaCl greatly enhances the mechanical performance of hydrogels. The fracture strain and fracture stress increase from 530% to 4500% and from 0.05 kPa to 0.71 kPa, respectively, as the content of NaCl is increased from 0.1 mmol/g to 3 mmol/g (Figure 2a,b). This is because the solubility of hydrophobic SMA in water is very low (10^−9^ mL/mL), and SMA is unable to enter SDS micelles through the continuous aqueous phase in the absence of salt [39]. Increasing NaCl content enhances the solubility of SMA and leads to the formation of micelle crosslinking points, thus producing a dense GG/HAP network. This inference is also confirmed by the FESEM images in Figure 1b,c.

The hydrogels without NaCl or with low NaCl content (such as AICH_0.5_) lose their flexibility when the temperature is decreased to −20 °C (Figure S2), because the formation of ice crystals limits the movement of network strands. However, a high content of NaCl (such as 3 mmol/g) greatly improves the stretchability of hydrogels at −20 °C (Figure S2). To quantitatively evaluate the mechanical property of AICH_3_ at subzero temperatures, the universal testing machine equipped with a low-temperature chamber is used for tensile measurement. Before testing, the sample was equilibrated in the chamber for 30 min. In contrast to AICH_0.1_, the AICH_3_ can be stretched to a strain of 1700% at −20 °C (Figure 2c,d). Moreover, the stress–strain curves of AICH_3_ at different subzero temperatures are shown in Figure 2e. With the decrease in temperature, the elastic modulus of AICH_3_ increases and the ductility decreases. However, the AICH_3_ can still be stretched to a strain of 830% even at −35 °C (Figure 2f).

**Figure 3:**
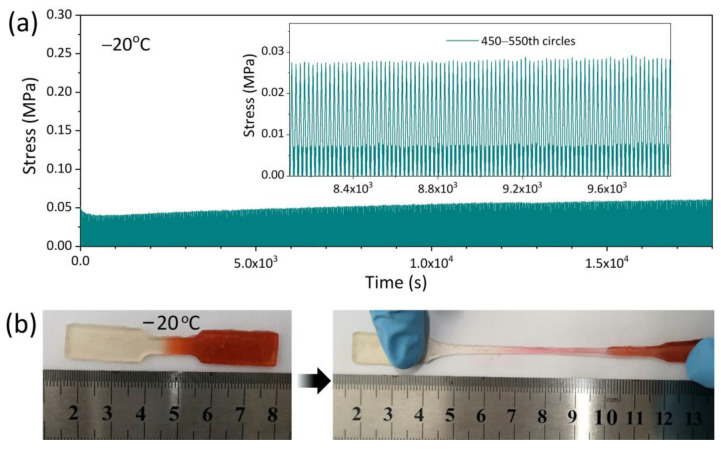
(**a**) The cyclic compressive tests of AICH_3_ at a constant strain of 20% at −20 °C. (**b**) The self−healing property of AICH_3_ at −20 °C.

The cyclic compressive tests with a strain of 20% were further performed at −20 °C to evaluate the anti-fatigue of AICH_3_ at subzero temperatures. It is found that the stress-strain curves basically remain constant over 1000 cycles (Figure 3a), suggesting that the AICH_3_ has an excellent anti-fatigue performance at subzero temperatures. In addition, the AICH_3_ also shows a self-healing capability at subzero temperatures. The self-healing experiment was carried out as follows: The dumbbell-shaped sample was cut into two pieces. Subsequently, the cut surfaces were brought together to form a contact and sealed into a polyethylene bag and then moved into the chamber of −20 °C and stored for 2 h. The cut sample was found to be well healed (Figure 3b), and the self-healed sample could be stretched to about four times of original length. The excellent anti-fatigue and self-healing capability of AICH_3_ at subzero temperatures is mainly attributed to the design of the physically crosslinked double network, where the ionic crosslinked GG network and HAP network synergistically contribute to improving the anti-fatigue and self-healing of AICH_3_ at subzero temperatures.

### 3.3. Conductivity and Strain Sensitivity of AICH

**Figure 4:**
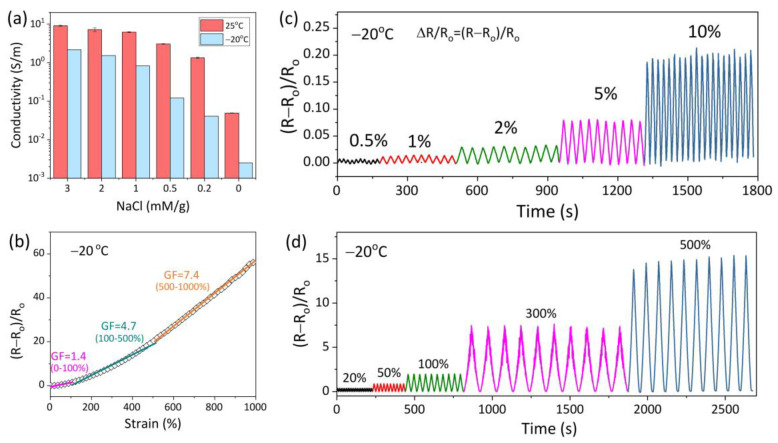
(**a**) Conductivity of AICH with various NaCl contents at 25 °C and −20 °C. (**b**) The change in GF of AICH_3_ with increasing tensile strain at −20 °C. The stability of AICH_3_ during cyclic loading-unloading with various tensile strains of (**c**) 0.5−10% and (**d**) 20−500%.

The presence of NaCl not only improves the mechanical property but also endows AICH_3_ with superior conductivity and strain-sensitivity at subzero temperatures. AICH_3_ achieves a conductivity of 2.2 S/m at −20 °C, which approaches 9.1 S/m at 25 °C (Figure 4a). In comparison, the conductivity of hydrogels with low NaCl content (<1 mM/g) at −20 °C is reduced by more than one order of magnitude than that obtained at 25 °C. The various conductivities of hydrogels with varying NaCl contents at subzero temperatures are mainly attributed to the freezing tolerance. For example, AICH_0.5_ has a freezing point of −18.5 °C, meaning that there are plenty of ice crystals formed at −20 °C, which greatly inhibits the movement of ions and results in an obvious decrease in conductivity. However, the freezing point of AICH_3_ is low to −43.6 °C, which ensures that there are plenty of free ions existing in the AICH_3_ at −20 °C, thus leading to an ignorable decrease in conductivity.

The strain sensitivity is usually studied through the relative resistance variation (∆R/R_0_ = (R − R_0_)/R_0_) under different applied strains. The gauge factor (GF), defined as the slope of ∆R/R_0_ vs. strain curve, is commonly employed to assess the strain sensitivity of a flexible conductor. Within tensile strain of 1000% (Figure 4b), the change in GF of AICH_3_ at −20 °C can be divided into three regions: (I) ε < 100%; (II) 100% < ε < 500%; (III) 500% < ε < 1000%, and the corresponding GF values are 1.4, 4.7 and 7.4, respectively. The increase in GF may be attributed to the fact that the ion migration channel becomes narrower and the migration path becomes longer when the AICH_3_ undergoes stretching deformation, thus resulting in the increase in ∆R/R_0_. It is noted that the conductivity and GF of AICH_3_ at subzero temperature overmatch the most reported conductive hydrogels [40,41,42,43,44,45,46] (Table 1).

In addition, the stable strain sensitivity is essential for the flexible strain sensors. To investigate the conductive stability, the AICH_3_ is cyclic loaded from tiny deformation (0.5% to 10%) to large strain (20% to 500%) at −20 °C (Figure 4c,d). It is apparent that the curves of ∆R/R_0_ versus time almost overlap at a given tensile strain, suggesting that AICH_3_ demonstrates an excellent repeatable and stable strain response in a wide range of strain (0.5–500%) at subzero temperatures, which ensures that AICH_3_ can be used to detect various deformations at subzero temperatures.

### 3.4. AICH_3_-Based Flexible Devices

**Figure 5:**
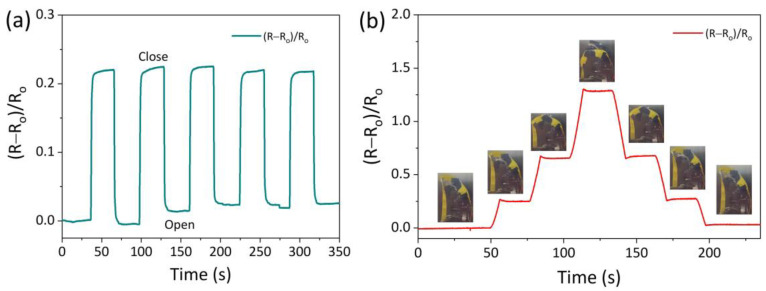
(**a**) The change in relative resistance when the AICH_3_-based strain sensor is fixed on the bionic finger and repeatedly bends at −20 °C, and (**b**) the change in relative resistance when the bending degree of the bionic finger gradually increases and then gradually recovers different bending degrees.

The outstanding mechanical property, conductivity, and strain sensitivity at subzero temperatures endow the AICH_3_-based strain sensor with the ability to monitor different deformations. As shown in Figure 5, the AICH_3_-based strain sensor was attached to the bionic finger and placed in the low-temperature chamber (−20 °C) to detect the bending deformation of the bionic finger. It can be seen that the AICH_3_-based strain sensor can accurately sense the tiny deformation of the bionic finger. Importantly, the resistance immediately fell back to the original level when the bionic finger was completely relaxed. At the same time, the AICH_3_-based strain sensor is capable of distinguishing the different bending angles of the bionic finger. When the bionic finger was held at a certain angle, the resistance almost remained at a constant value and returned to the original value after straightening the finger. These results indicate that the AICH_3_ has potential for fabricating the wearable electronic skin.

**Figure 6:**
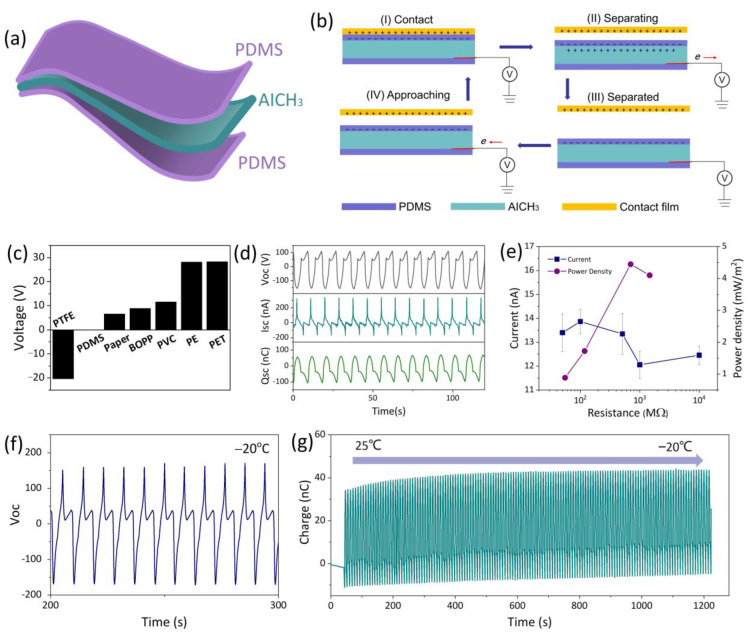
(**a**) Scheme of AICH_3_-based TENG. (**b**) Working mechanism of AICH_3_-based TENG. (**c**) Comparison of output voltage as the various contact materials are used. (**d**) The open-circuit voltage (Voc) in black color, short-circuit current (Isc) in dark cyan color and short-circuit charge in olive-green color (Qsc) of AICH_3_-based TENG. (**e**) Instantaneous current and area power density of AICH_3_-based TENG at various resistances. (**f**) The Voc of AICH_3_-based TENG at −20 °C. (**g**) The Qsc of AICH_3_-based TENG changes as the temperature decreases from 25 °C to −20 °C.

Recently, triboelectric nanogenerators (TENGs) have been rapidly developed for converting mechanical energy into electrical energy. Herein, the AICH_3_ is utilized as an electrode to fabricate a TENG with a single-electrode working mode (Figure 6a). When a material touches PDMS, an equal number of positive and negative charges are generated at the interfaces (Figure 6b, I), owing to the difference in electronic affinity between PDMS and the contact material. When the contact material is separated from PDMS, the negative charges at the surface of PDMS induce the migration of ions in AICH_3_ owing to the electrostatic equilibrium, leading to electrons flowing from the AICH_3_ electrode to ground and the generation of electric current (Figure 6b, II). When the contact material and PDMS are separated completely, a new electrostatic equilibrium is established (Figure 6b, III). Once the contact material is back close to PDMS, electrons flow back from ground to AICH_3_, generating a reverse current (Figure 6b, IV). Repeating the contact–separation process will generate a detectable alternating current. In contrast to traditional hydrogels, the AICH_3_ utilized as an electrode will greatly extend the application range of TENG owing to its excellent conductivity at subzero temperatures.

The output performance of AICH_3_-based TENG using commercial PET, PE, PVC, PTFE, BOPP, and paper as contact materials (length × width = 3 cm × 4 cm) is evaluated. Due to the different electronic affinities between PDMS and contact materials, the AICH_3_-based TENG can output various electrical signals. Among them, the PET film used as contact material produced a maximum output voltage (Figure 6c). Therefore, in the following quantitative evaluation, the PET film is used as the contact material to measure the output performance of AICH_3_-based TENG. As shown in Figure 6d, the peak values on the open-circuit voltage (Voc), short-circuit current (Isc) and short-circuit charge (Qsc) are 111 V, 324 nA and 161 nC, respectively. Through loading external resistances from 50 MΩ to 10 GΩ, the output power of the AICH_3_-based TENG is obtained. When the resistance is 1 GΩ, the output areal power density reaches a maximum value of 4.43 mW/m^2^. In addition, the output performance of the AICH_3_-based TENG at subzero temperatures is also evaluated. It is found that the Voc of AICH_3_-based TENG almost maintains a constant value at −20 °C (Figure 6f). Decreasing the temperature from 25 °C to −20 °C shows a negligible influence on the Isc. These results indicate that the AICH_3_-based TENG has an outstanding and stable energy conversion efficiency, even at subzero temperatures.

**Figure 7:**
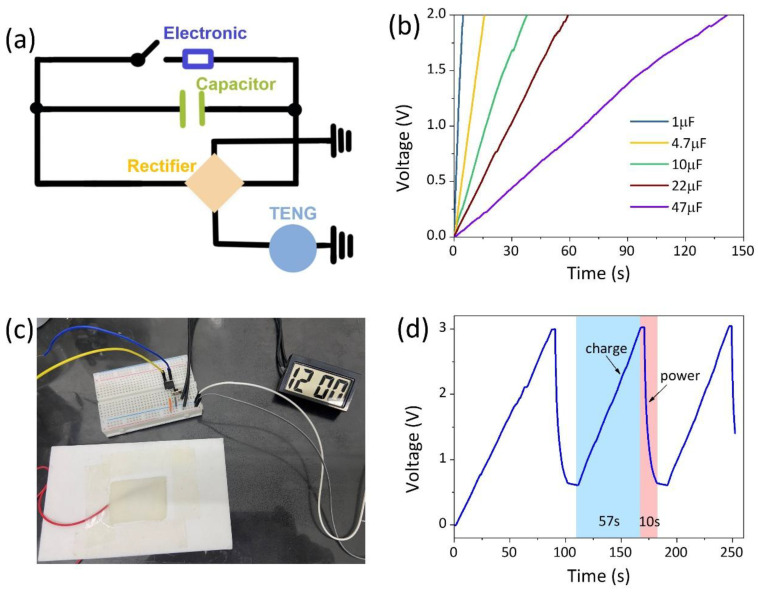
(**a**) The equivalent circuit and (**c**) the photograph of a self-charging power system. (**b**) Charging curves of various commercial capacitors using the AICH_3_-based TENG as the power source. (**d**) Voltage profile when the commercial capacitor with a capacity of 22 μF is charged by the AICH_3_-based TENG and powers a digital watch.

To evaluate the potential application of AICH_3_-based TENG in energy harvesting, a self-charging power system was designed. The equivalent circuit is shown in Figure 7a; it is composed of an electronic, a commercial capacitor, a bridge rectifier, and the AICH_3_-based TENG. The capacitor with varied capacitance values is charged by the AICH_3_-based TENG. The capacitor of 1 μF can be charged to 2.0 V within 5 s, but the charging rate decreases with the increase in capacitance value (Figure 7b). To demonstrate the performance of energy harvesting, a digital watch is connected to the circuit to form a self-charging and powering circuit (Figure 7c). The commercial capacitor with the capacity of 22 μF is charged to a voltage of 3 V within 57 s and can power the digital watch for about 10 s (Figure 7d). Furthermore, the capacitor can be charged and discharged repeatedly to power the digital watch.

## 4. Conclusions

In this work, we synthesized an anti-freezing ionic conductive hydrogel with a fracture stress of 1.1 MPa, fracture strain of 1700%, conductivity of 2.2 S/m, and gauge factor of 7.4 at −20 °C. The excellent mechanical, conductive, and strain-sensitive properties at subzero temperatures are mainly attributed to the multifunctionality of Na^+^. That is to say, the presence of Na^+^ not only promotes the construction of a dynamic crosslinked GG/HAP double network but also inhibits the formation of ice crystals at subzero temperatures. Benefiting from excellent mechanics, conductivity, and strain sensitivity, the assembled AICH_3_-based strain sensor can accurately detect the bending of the bionic finger at subzero temperatures. In addition, the AICH_3_ can also be used as a stretchable electrode, and the assembled AICH_3_-based TENG can effectively harvest energy and power electronic devices at −20 °C. Overall, this work provides a feasible strategy for preparing anti-freezing ionic conductive hydrogel and expands the application of hydrogels at subzero temperatures.

## Figures and Tables

**Table 1:** Comparison of the conductivity and GF for the AICH_3_ and the reported ionic conductive gel at subzero temperatures.

References	Temperature (°C)	Conductivity (S/m)	GF at Strain Range
This work	−20	2.2	1.4 at 0–100%
This work	−20	2.2	4.7 at 100–500%
This work	−20	2.2	7.4 at 500–1000%
Ref. [40]	−20	0.01	3.9 at 0–280%
Ref. [41]	−20	0.32	-
Ref. [42]	−70	1.1	1.2 at 120%
Ref. [43]	−40	2.7	-
Ref. [44]	−18	1.9	1.9 at 0–200%
Ref. [45]	−20	0.1	1.5 at 0–150%
Ref. [46]	−30	0.32	0.6 at 0–100%

## Data Availability

Data are contained within the article or Supplementary Material.

## Supplementary Materials

The following supporting information can be downloaded at: https://www.mdpi.com/article/10.3390/polym17233102/s1, Figure S1: The relationship of phase angle (tanδ) with temperature; Figure S2: The photographs of tensile tents of AICH_0.5_ and AICH_3_ at −20 °C.
