# Supplementary material for: An Anti-Freezing Ionic Conductive Hydrogel for Strain Sensing and Energy Harvesting Devices
Source: Polymers (Basel). 2025 Nov 22;17(23):3102. doi: 10.3390/polym17233102 (PMC12694472; doi:10.3390/polym17233102)
Supplement: Supplementary file 1 [file polymers-17-03102-s001.zip › polymers-3939615-supplementary.pdf]

Supporting Information

An Anti-Freezing Ionic Conductive Hydrogel for Strain Sensing and Energy Harvesting Devices

Yanjie Wang, Wei Yu * and Sijun Liu *

Advanced Rheology Institute, Department of Polymer Science and Engineering, Shanghai Jiao Tong University, Shanghai 200240, China; yanjiewang@sjtu.edu.cn

* Correspondence: wyu@sjtu.edu.cn (W.Y.); liusijun@sjtu.edu.cn (S.L.)

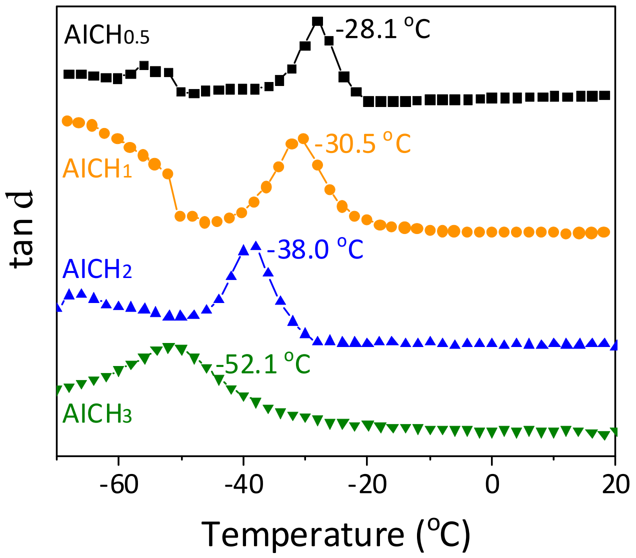

Figure S1. The relationship of phase angle (tanδ) with temperature.

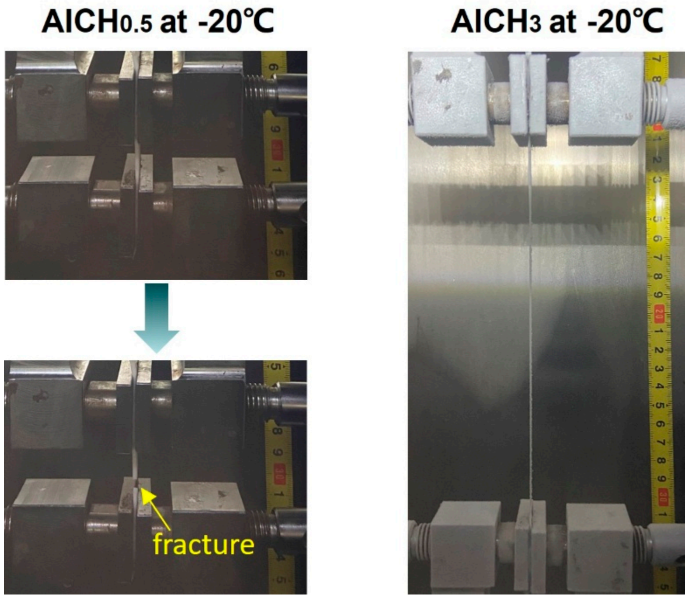

Figure S2. The photographs of tensile tents of $AICH_{0.5}$ and $AICH_3$ at –20 °C.
